# A Randomized Double Blind Placebo Controlled Trial Examining the Effects of Pentoxifylline on Contrast Induced Nephropathy Reduction after Percutaneous Coronary Intervention in High Risk Candidates

**DOI:** 10.22037/ijpr.2019.12557.10977

**Published:** 2019

**Authors:** Farnaz Barzi, Reza Miri, Roxana Sadeghi, Mohammad Sistanizad, Mohsen Sadeghi, Mohammad Parsa Mahjoob, Mohammad Chehrazi

**Affiliations:** a *Department of Internal Medicine, School of Medicine, Shahid Beheshti University of Medical Sciences, Tehran, Iran. *; b *Cardiovascular Research Center, School of Medicine, Shahid Beheshti University of Medical Sciences, Tehran, Iran.*; c *Department of Clinical Pharmacy, School of Pharmacy, Shahid Beheshti University of Medical Sciences, Tehran, Iran. *; d *Department of Biostatistics and Epidemiology, School of Medicine, Babol University of Medical Sciences, Babol, Iran. *

**Keywords:** Pentoxifylline, Contrast-induced nephropathy, Angiography, Mehran score, Acute kidney injury

## Abstract

Contrast-induced nephropathy (CIN) (known as contrast-induced acute kidney injury) occurs as a result of acute worsening of renal function following a procedure with administration of iodine contrasts agent and remains a substantial concern in clinical practices. The purpose of this study is to investigate the preventive effect of Pentoxifylline supplementation on reduction of CIN occurrence after percutaneous coronary intervention among patients who were high risk of CIN according to Mehran score. In randomized, double-blind clinical trial patients who undergo coronary angiography with Mehran Score ≥ 11 consisted of our population. Patients in a ratio 1:1, divided into two groups received saline 0.9% plus N-acetyl cysteine and Pentoxifylline 400 mg three times per day 24 h before angiography until 48 h after angiography. In control group, the patients received placebo instead of PTX in a same manner as the control group. The endpoint was the incidence of CIN defined as creatinine increase of 0.5 mg/dL within 2 days after contrast. There were no significant differences in baseline characteristics. CIN occurred in 3 (5.5%) and 4 (7.3%) patients of the both groups (Pentoxifylline and control), respectively (*p = *0.69; incidence odds ratio 1.36; 95% CI 0.29-6.38). No significant differences were seen in secondary outcome measures and changes in the level of creatinine (*p = *0.54). In high-risk patients undergoing coronary angiography pentoxifylline supplementation had protection effect against contrast-induced nephropathy greater than placebo based hydration, but, not supported by our data.

## Introduction

Contrast-induced nephropathy (CIN) (known as contrast-induced acute kidney injury) occurs as a result of acute worsening of renal function following a procedure with administration of iodine contrasts agent and remains a substantial concern in clinical practices. Generally, the incidence of CIN is 2%, but it is higher in at-risk groups of patients who have both chronic kidney disease (CKD) defined as an estimated glomerular filtration rate (eGFR) <60 mL/min/1.73 m^2^ and diabetes mellitus (1-5). The incidence of CIN is as high as 50% for patients with multiple risk factors including renal insufficiency, age more than 70 years17, female gender18, BMI > 25, smoking, congestive heart failure, and using nephrotoxic medications and dehydration with hypotension (6-8). CIN contributes to the elongation of hospital stays and may lead to permanent reduction in renal function or even to increased mortality and end stage renal disease (9).

Though various treatment strategies aimed at preventing CIN were tested in the past, only adequate oral and/or intravascular hydration and use of lowest possible volume of a low- or iso-osmolar contrast agent have been commonly recommended in most recent guidelines (10-12). Despite the recent attempts that aim to control certain risk factors to prevent CIN occurence in the postoperative period of cardiac surgery, the incidence of renal dysfunction remains high, especially in high risk-patients (9, 13-15). For this reason, there is a need to seek more effective alternatives to prevent this complication. Some medications such as, N-acetyl cysteine (NAC), statins, theophylline, and vitamin E or C have been used to reduce the incidence of CIN. In this regard, Pentoxifylline (PTX) would have preventive effects on CIN through facilitating RBC passage from vessels by increasing its elasticity, decreasing blood viscosity, reducing platelet aggregation, and thrombus formation. Moreover, it has anti-inflammatory and anti-oxidative effects that may be significant in prevention of CIN (16-18). 

Therefore, the purpose of this randomized control study was to investigate the effect of Pentoxifylline supplementation on reduction of CIN occurrence after percutaneous coronary intervention among patients who were high risk of CIN according to Mehran score. Mehran *et al.* have introduced a simple risk score system for prediction of CIN. They classified the patients based on the risk scores to the mild, moderate, and high-risk. This is the first time to investigate such a preventive effect among high risk patients.

## Experimental

*Patient population*

The patients who were hospitalized in the Clinics of Diabetology or Cardiology of Imam Hossien hospital between January 1, 2014 and August 27, 2014 for an elective radiologic procedure with contrast medium use were assessed for eligibility into the study. Inclusion criterion was only Mehran score more than or equal to 11. Exclusion criteria included need for urgent coronary angiography in the first 48 h after admission, history of contrast media usage in previous 14 days or renal failure in recent six months, multiple myeloma, pregnancy or breastfeeding and pre-planned use of any other measure for CIN prevention apart from PTX consumption.

The study protocol was approved by the Ethical Committee of Shahid Beheshti University of Medical Sciences. All patients gave written consent prior to study entry. This study was registered in irct.ir, and the registered number is IRCT2016111530905N1.

**Table 1:** Demographic and clinical data of enrolled patients at baseline

	**PTX+NAC**	**Placebo+NAC**	***p*** **-value**
Age (years)	70.10 (11.80)	68.30 (10.83)	0.41
Gender-M/F (No.)	23/32	29/26	0.25
DM-no/yes (No.)	26/29	21/34	0.24
ACEIs/ARB (No.)	28/27	27/28	1.00
Statins-no/yes (No.)	11/44	12/43	0.81
Theophylline- no/yes (No.)	54/1	55/0	0.31
Vit E or C- no/yes (No.)	55/0	54/1	0.31
HTN- no/yes (No.)	15/40	21/34	0.22
Smoking- no/yes (No.)	49/6	46/9	0.40
NSAIDs- no/yes (No.)	51/4	51/4	1.00
Diuretics- no/yes (No.)	42/13	37/18	0.28

Mean (SD); DM: Diabetes Mellitus; ACEIs: Angiotensin converting enzyme inhibitors; ARB: Angiotensin receptor blocker; NSAIDs: Non- steroid anti-inflammatory drug; HTN: Hypertension; Vit: Vitamin.

**Table 2:** Level of serum Creatinine before, after and changes (Δ) post-coronary angiography in the PTX supplementation and control groups

	**PTX+NAC**	**Placebo+NAC**	***p*** **-value**
Cr (µmol/L) (before)	1.31 (0.81)	1.20 (0.33)	0.33
Cr (µmol/L) (after)	1.34 (1.04)	1.19 (0.36)	0.31
Δ Cr (µmol/L)	0.03 (0.35)	-0.006 (0.24)	0.54
CIN-yes/no (No.)	2/53	2/53	

Mean (SD); Cr: Creatinine; PTX: Pentoxifylline.

**Figure 1:**
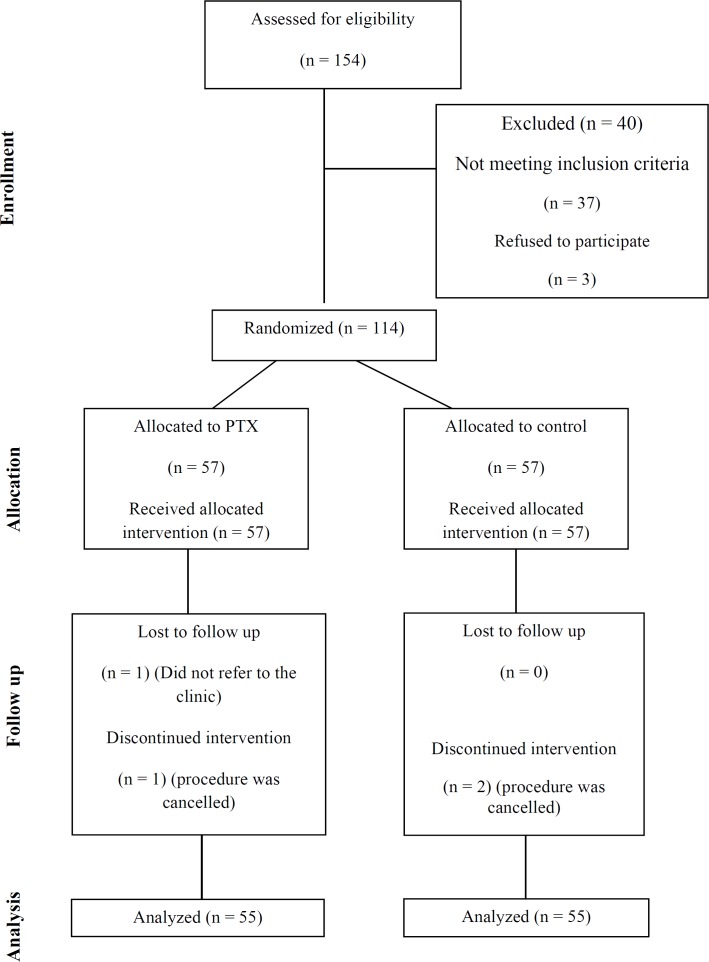
Flow of study participants through the stages of the trial; CONSORT 2010 Flow Diagram

*Study protocol*

One hundred ten eligible patients (women and men) sequentially were enrolled to this study and assigned to one of two groups by the hospital pharmacy staff otherwise not involved in the study based on a computer-generated randomization schedule with the use of numbered opaque envelopes containing the identification of the assigned medication. After opening the envelope with the serial number of the patient in the study, the pharmacy staff prepared one bottle containing 400 mg of assigned capsules which was marked only by the serial number and name of the patient for three times per day from 24 h before to 48 h after angiography. The patients in the control group received placebo capsules in the same shape as PTX. We gave saline 0.9% 0.5-1 mL/kg/hour based on kidney function and heart status from 12 h before until 12 h after angiography to maintain urine volume more than 150 mL/hour.

All patients in both groups received intravenous hydration with 0.9% saline plus NAC. We administered NAC to all patients with the same dose of 1200 mg every 12 h from 24 h before to 48 h after angiography. None of the patients in either group received sodium bicarbonate before coronary angiography. Pre-procedural consumptive medications were recorded including, Angiotensin-converting enzyme inhibitor (ACEI), Angiotensin receptor blocker (ARB), diuretics, statin, theophylline, vitamin E and C. Medications with nephrotoxic effects, including Metformin and non-steroid anti-inflammatory drugs (NSAIDs) were discontinued for 72 h during angiography. All patients in both groups received the same contrast media Visipaque (iodixanol) 320, GE Healthcare, Cork, Ireland. Serum creatinine level was measured before and 48 h after coronary angiography with one technique in the same laboratory. eGFR was calculated with MDRD (Modification of Diet in Renal Disease) formula (19). Primary end-point was the occurrence of CIN defined as an increase in serum creatinine ≥ 0.5 mg/ dL from baseline to 48 h after angiography.

*Statistical Analysis*

Continuous and categorical variables are expressed as mean (standard deviation) and frequency. Normality of the quantitative data was evaluated by Kolmogorov-Smirnov test. The two sample *t*-test on differences between pre-post measures and chi-square or Fischer’s exact test in appropriate were used for continuous and categorical variables, respectively. *p*-value < 0.05 was considered statistically significant. The analyses were performed with SPSS software version 21 (SPSS Inc., Chicago IL, USA).

## Results

During the study period, 154 patients were assessed for eligibility, 40 of whom were excluded for either not meeting inclusion criteria or unwillingness to participate. Of the 114 patients randomized, 110 (PTX+NAC group, n = 55; placebo+NAC group, n = 55) subjects who had undergone all study procedures and had at least one post-intervention creatinine available were included into the analysis. Two patients in each group were excluded from analysis due to subsequent withdrawal of consent or cancelation of the contrast-requiring examination/intervention (Figure 1 – Study Flow Diagram).

Table 1 shows demographic and clinical characteristics in two treated groups at baseline, demonstrating both groups were comparable with regard to gender distribution, age, angiotensin converting enzyme inhibitors, angiotensin receptor blocker, Non-steroid anti-inflammatory drug, hypertension, smoking, theophylline, statin, diabetes mellitus (DM), diuretics, and consuming vitamin E or C. 

No significant difference was seen between both groups regarding CHF. Fourty-five patients in PTX group and 44 patients from the control group had CHF (*p *> 0.99) type and volume of contrast were same between both groups and patients. 

The primary outcome event, CIN, occurred in 3 (5.5%) and 4 (7.3%) patients of the PTX and control groups, respectively, with a non-significant difference between the groups (*p *= 0.69; incidence odds ratio 1.36, 95% confidence interval 0.29-6.38). Similarly, no significant differences were seen in any of the secondary outcome variables (Table 2).

Mean serum creatinine level was 1.31 (0.81) mg/dL and 1.20 (0.33) mg/dL in PTX and control groups, respectively (*p = *0.33). Fourty-eight h after angiography, the mean serum creatinine level was 1.34 (1.04) mg/dL and 1.19 (0.31) mg/dL in PTX and control groups, respectively. There were no significant changes between both groups regarding mean serum creatinine levels (*p = *0.54). We did not see any complication due to PTX usage. Besides, no patient died or experienced severe kidney injury with need for acute dialysis treatment. At the time of follow-up inquiry, 1 patient in the PTX group and 2 patients in the control group had reached ESRD with need for chronic dialysis treatment. All three patients had had already advanced chronic kidney failure at the time of the contrast-requiring procedure. One patient in the PTX group had been lost to follow-up.

## Discussion

PTX is a methylxanthine-derivative drug that has been used for many years in the treatment of peripheral vascular disease. PTX is also a vigorous suppressor of tumor necrosis factor-alpha (TNF-alpha) secretion and has indicated affect in the treatment of defined human and animal inflammatory diseases (20). In the recent study, we found that using pentoxifylline in addition to good hydration and NAC cannot decrease the incidence rate of contrast nephropathy in the high-risk group of patients who were undergoing coronary angiography. In a study by Firouzi *et al.*, which was performed on patients who underwent coronary angioplasty, CIN occurred in 13.69% of patients in control group versus 8.5% in the PTX group (17). The observed difference was not statistically significant (*p = *0.17). However, the authors recommended that the prophylactic use of PTX could be recommended for CIN prevention before coronary angioplasty (17). Incidence rate of CIN in their study was investigated on all patients without risk stratification that underwent coronary angioplasty. In the recent study, the incidence of CIN in high-risk groups of patients (high Mehran’s score) was similar to studies who enrolled low-risk population. We reached a lower incidence rate of CIN. According to Mehran score, we select high-risk group of patients underwent coronary angiography. The incidence rate was 5.5% and 7.3 for PTX and control group, respectively. The lower incidence of CIN may be contributed to the low volume of contrast usage (less than 50 mL) as our studied population underwent coronary angiography not angioplasty. In a study by Marenzi *et al.* CIN occurred in 19% of patients underwent percutaneous coronary intervention (PCI) after myocardial infarction. The researchers emphasized on the high rate of CIN after PCI especially in the high-risk group of patients and even in the patients who had normal renal function (21). Among prophylactic strategies in patients who are moderate to high risk for CIN, hydration is the best and proven way to prevent nephropathy (11). We tightly controlled the urine output by good hydration, which was not emphasized in study by and would be the other reason of lower incidence rate of CIN in our study. One of the medications could imply preventive effect on CIN is N-acetylcysteine (NAC) (14, 17 and 22). We gave NAC in addition to good hydration to both study and control groups of the study with the same dose which was the different preventive approach from studies by Firouzi *et al.* and Yavari *et al.* (17, 18). Some medications may have a beneficial role in prevention of CIN such as statins, theophylline, and vitamin C (23-26). Although a recent meta-analysis showed a beneficial effect of high-dose atorvastatin for prevention of CIN in our study, there was no significant difference between study and control groups regarding consumption of these agents (15). One of the limitations in our study is using serum creatinine level to define CIN. Based on some studies an absolute increase in serum creatinine is a good threshold for the diagnosis of CIN (27, 28). Increasing in serum creatinine is relatively slow, and it takes at least 48 to reach a level of nephropathy; however, using biomarker of kidney function that are more rapidly rising and are the precise and sooner markers, which better indicate to nephropathy such as cystatin C, allow more accurate estimation of CIN (29). We propose to accomplish large size of study population with moderate and high-risk of CIN who underwent coronary angioplasty, which would reach us to better results according to the supplementation effects of PTX.

## Conclusion

In conclusion, our results do not support the preferential use of PTX for the prevention of contrast-induced nephropathy in high-risk patients. On the other word, the patients undergoing coronary angiography PTX supplementation had protection effect against contrast-induced nephropathy greater than placebo based hydration, but, not supported by our data. However, it would be recommended to run a multicenter study in high risk patients.
